# Human Papilloma Virus and Cancer Stem Cell markers in Oral Epithelial Dysplasia—An Immunohistochemical Study

**DOI:** 10.5041/RMMJ.10451

**Published:** 2021-10-25

**Authors:** Prasanth Thankappan, Madhavan Nirmal Ramadoss, Tharmasahayam Isaac Joseph, Percy Ida Augustine, Isaacjoseph Bevin Shaga, Jashree Thilak

**Affiliations:** 1 Department of Oral and Maxillofacial Pathology, Sree Mookambika Institute of Dental Sciences, Kulasekharam, Kanyakumari District, Tamil Nadu, India; 2 Department of Oral and Maxillofacial Pathology, Rajah Muthiah Dental College and Hospital, Annamalai University, Chidambaram, Tamil Nadu, India; 3 Department of Orthodontics and Dentofacial Orthopedics, Rajas Dental College, Tirunelveli District, Tamil Nadu, India; 4 International Cancer Center, Neyyoor, Kanyakumari District, Tamil Nadu, India

**Keywords:** ALDH1, CD44, OCT4, oral epithelial dysplasia, p16, SOX2

## Abstract

**Objectives:**

To study the correlation between the putative cancer stem cell (CSC) markers aldehyde dehydrogenase 1 (ALDH1), cluster of differentiation 44 (CD44), sex-determining region Y-box 2 (SOX2), and octamer-binding protein 4 (OCT4) and human papilloma virus (HPV) infection using p16, the surrogate marker of HPV in oral epithelial dysplasia (OED) and normal mucosa.

**Methods:**

Five sections each from 40 histopathologically diagnosed cases of different grades of OED and 10 cases of normal oral mucosa without dysplasia were immunohistochemically stained with p16, ALDH1, CD44, SOX2, and OCT4, respectively.

**Results:**

Expression of ALDH1 and SOX2 was significantly increased in OED cases, whereas CD44 and OCT4 expression was increased in normal mucosa. P16-positive OED cases showed upregulation of ALDH1 and OCT4 expression as compared to p16-negative cases, while CD44 and SOX2 expression was downregulated in p16-positive OED cases; however, the results were not statistically significant.

**Conclusion:**

The present study indicated a suggestive link between p16 and cancer stem cell marker expression in HPV-associated OED, and that p16 has a significant role in CSC progression in OED. This is the first study to evaluate the expression of putative CSC markers in HPV-associated OED. However, low study numbers are a potential limiting factor in this study.

## Introduction

Head and neck squamous cell carcinoma (HNSCC) is one of the leading causes of cancer death worldwide, with 95% of cases being oral squamous cell carcinoma (OSCC).^1^ Oral squamous cell carcinoma is often preceded by potentially malignant disorders of the oral mucosa with an unpredictable course of progression.^2^ The presence of epithelial dysplasia in potentially malignant oral disorders is generally regarded as one of the most significant predictors of malignant transformation.^3^ Various studies reported a malignant transformation rate in the range 10.5%–12.1% amongst patients with histologically confirmed oral epithelial dysplasia (OED) undergoing long-term follow-up.^4^

Even though tobacco and alcohol use are the primary risk factors for HNSCC, 25% to 35% of cases have been shown to be associated with human papillomavirus (HPV).^5^,^6^ Patients with HPV-positive oropharyngeal tumors have distinct clinical features and a more favorable prognosis compared to those with HPV-negative tumors.^6^ The reported prevalence of HPV in OSCC varies from 4% to 95%.^7^ Due to contradictory reports on confirmed HPV prevalence, the role of HPV in the progression of oral dysplasia has remained a point of contention. Although progression characteristics are not fully elucidated, this HPV-associated oral dysplasia could potentially progress into HPV-associated OSCC.^8^,^9^

Overexpression of the p16INK4a protein can act as a surrogate biomarker of HPV-induced carcinomas.^10^ Under normal circumstances, the p16 protein can inhibit cell cycle progression by restraining the retinoblastoma protein phosphorylation during the gap 1 and synthetic phases of the cell cycle. Loss of p16 due to hypermethylation of the promoter region, homozygous deletion, or loss of heterozygosity is commonly found in OSCC patients with conventional risk factors. Moreover, loss of p16 function can be found in potentially malignant disorders such as oral leukoplakia and erythroplakia, suggesting a role for p16 in the early stage of carcinogenesis. On the other hand, as previously mentioned, p16 is aberrantly overexpressed in patients with high-risk HPV-associated HNSCC. Taking advantage of this phenomenon, p16 is widely used as a surrogate marker for HPV-related HNSCC.^11^ Positive p16INK4a immunostaining of HPV-associated tumors is 100% sensitive but only 79% specific.^12^

Accumulating evidence indicates that the initiation, progression, recurrence, and metastasis of HNSCC are related to the behavior of a small subpopulation of cells known as cancer stem cells (CSCs). The CSCs can be identified and isolated by the expression of distinctive markers to enrich stem cells.^13^–^15^ Various CSC markers such as cluster of differentiation 44 (CD44), aldehyde dehydrogenase 1 (ALDH1), sex-determining region Y (SRY)-box 2 (SOX2), and octamer-binding protein 4 (OCT4) have been utilized to identify CSCs in HNSCC and OED.^16^

Aldehyde dehydrogenase 1 (ALDH1) is a cytosolic detoxifying isoenzyme that oxidizes intracellular aldehydes and thus contributes to the oxidation of retinol, resulting in retinoic acid in early stem cell differentiation. This is required for the maintenance of its self-renewing property. It is not only a potential marker of “stemness,” but it also plays a role in the biology of tumor-initiating cells.^17^

Cluster of differentiation 44 (CD44) is a multifunctional transmembrane glycoprotein expressed in many types of cancer. This marker interferes with the intercellular binding, migration, and angiogenesis of cancer cells.^18^

The SOX2 protein is a high-mobility SRY-related group box transcription factor; SOX2 is involved in multiple signal transduction pathways in normal developmental and many pathological processes including cell proliferation, migration, invasion, stemness, tumorigenesis, anti-apoptosis, and chemoresistance.^19^–^21^

The transcription factor OCT4 is a regulator of the Pit-Oct-Unc domain and is critical in early embryogenesis and maintenance of embryonic stem cell pluripotency.^22^ Octamer-binding protein 4 (OCT4) has also been linked to oncogenic processes, and it has been suggested that OCT4 plays a role in tumor transformation, tumorigenicity, invasion, and metastasis within OSCC.^23^,^24^ It has also been proposed that OCT4 promotes tumor initiation by playing a role in the regulation of epithelial-mesenchymal transition.^25^

This present study aims to investigate the immunoexpression of these putative CSC markers in OED and its correlation with the HPV status identified using p16 immunostaining—the surrogate marker for HPV. Moreover, a limited number of studies have compared the CSC markers and HPV infection in OED. Thus, determining if there is an association between HPV infection and CSC marker expression in OED would provide a better understanding regarding the significance of CSCs in HPV-positive OED.

## MATERIALS AND METHODS

### Ethical Clearance and Sampling

This was a retrospective study of biopsy specimens of oral mucosal lesions submitted to the Department of Oral and Maxillofacial Pathology, Sree Mookambika Institute of Dental Sciences, Kanyakumari, India. This study was approved by the Institutional Human Ethics Committee, and all specimens were processed following the World Medical Association Declaration of Helsinki (version 2008) and according to the Indian Council of Medical Research Guidelines regarding the use of human tissues. Each individual in this study signed and approved the informed consent before biopsy. Fifty formalin-fixed paraffin-embedded (FFPE) tissue blocks were used for the study: these blocks consisted of 40 cases of varying grades of oral epithelial dysplasia (OED Group) reported in our institution in 2019 and 10 samples of normal mucosa (Normal Group). The sample size was calculated with a 95% confidence interval and 10%–20% relative precision. Histopathologic assessment of all clinically diagnosed premalignant lesions for the degree of epithelial dysplasia was performed according to the 2017 WHO classification;^26^ OED grades were mild dysplasia (*n*=7), moderate dysplasia (*n*=13), and severe dysplasia (*n*=20). Non-inflamed tissue samples acquired during dental surgical procedures were used for the normal tissue.

### Immunohistochemistry Procedure

The immunohistochemistry (IHC) studies for tissue samples were performed using the following ready-to-use antibodies (Master Diagnostica, Vitro, Spain): goat anti-human ALDH1A1 polyclonal (MAD-000611QD-R-3) (ALDH1), rabbit anti-human CD44 monoclonal (MAD-000537QD-R-3) (CD44), rabbit anti-human SOX2 monoclonal (MAD-000521QD-R-3) (SOX2), mouse anti-OCT3/4 monoclonal (MAD-000239QD-R-3) (OCT4), and mouse anti-human p16INK4a monoclonal antibody (MAD-000690QD-R-3) (p16). The IHC recognition of these markers was performed using the Dako Antibody Detection System (Agilent Technologies, Santa Clara, CA, USA).

Each FFPE tissue block was cut into five 4-μm thick sections, placed on positively charged slides (FLEX IHC Slides K802021; Agilent, Santa Clara, CA, USA), and stained for ALDH1, CD44, SOX2, OCT4, and p16 antibodies, respectively. Briefly, the 4-μm thick paraffin-embedded tissue sections were deparaffinized in xylene and rehydrated in graded alcohols and distilled water. Antigens were retrieved in target retrieval solution high-pH Tris/ethylenediaminetetraacetic acid buffer (pH 9), using a heat-induced epitope retrieval method at 95°C for 40 min. The slides were then washed twice with Tris buffer. To block endogenous peroxidase activity, sections were incubated for 30 min in ready-to-use peroxidase-blocking reagent in a moist chamber, followed by twice washing with Tris buffer. The IHC staining was performed at 37°C for 30 min in a moist chamber. Secondary antibodies conjugated with horseradish peroxidase were applied at room temperature for 30 min. After washing twice with Tris buffer, the sections were incubated with diaminobenzidine solution for 10 min and microscopically observed for color development. The sections were counterstained with Harris hematoxylin for 1 min, washed with tap water for 1 min, air dried, cleared with xylene, coverslipped using dibutyl phthalate, and examined by light microscopy. Positive controls for ALDH1, CD44, SOX2, OCT4, and p16 immunostaining were performed in stomach, tonsil, normal skin, seminoma, and cervical dysplasia tissues, respectively. Negative controls were obtained by omitting the primary antibodies. A semiquantitative assessment of antigen expression in the cells was performed using a Labomed Lx500 light microscope (Labomed Inc., Los Angeles, CA, USA). Expression in the epithelial cells was considered positive when dark brown staining was observed in SOX2 and OCT4 in the nuclei, CD44 on the membrane, ALDH1 in the cytoplasm, and p16 in both the nuclei and the cytoplasm. All slides were evaluated independently by two observers.

### Staining Assessment

Positive ALDH1, CD44, SOX2, and OCT4 immunoexpression for different OED grades was scored as described below. Five random fields were observed under ×400 magnification, and the sections were scored 0–3 for staining intensity as follows: 0, no stain; 1, pale brown; 2, brown; and 3, dark brown. The percentage of positively stained epithelial cells were scored as: 0 (0%), 1 (<25%), 2 (25%–49%), 3 (50%–74%), and 4 (75%–100%), and calculated as follows:

$$Percentage\;of\;Positive\;Cells=\frac{Number\;of\;Positive\;Cells}{Total\;Number\;of\;Counted\;Cells}\times 100$$

The final index score, which ranged from 0 to 12, was the product of the labeled percentage positive score and stain intensity score. Specimens were divided into two groups based on their overall scores: low expression, <2 points; high expression, ≥2 points.

### Human Papilloma Virus Status

To evaluate p16, cases were scored as positive when a diffuse pattern of nuclear and cytoplasmic staining was seen in dysplastic epithelial cells, involving at least half of the epithelial thickness. Cases were scored as negative when no staining was present, or if scattered single cells were positive within the dysplastic epithelium, a commonly used HPV detection methodology.^27^–^29^

### Statistical Analysis

Data were expressed as number, percentage, mean, and standard deviation (SD). Statistical Package for Social Sciences (SPSS) version 20.0 was used for analysis. Unpaired *t* test and chi-square test were used, and a *P* value less than 0.05 was considered statistically significant at a 95% confidence interval.

## RESULTS

### Demographic Findings

Demographic details and clinicopathological parameters of patients and controls are shown in Table 1.

**Table 1:** Demographic Details and Clinicopathological Parameters of OED Group versus Normal Group.

Parameters	OED Group (*n*=40)	Normal Group (*n*=10)
Age (years)		
40–50	4 (10%)	6 (60%)
51–60	13 (32.5%)	4 (40%)
61–70	16 (40%)	0
71–80	7 (17.5%)	0

Gender		
Male	35 (87.5%)	7 (70%)
Female	5 (12.5%)	3 (30%)

Habits		
Betel nut	12 (30%)	0
Chewing tobacco	10 (25%)	0
Smoking tobacco	18 (45%)	0
Alcohol consumption	35 (87.5%)	0

Clinical Appearance		Not Applicable
Erythroleukoplakia	11 (27.5%)	
Erythroplakia	8 (20%)	
Leukoplakia	15 (37.5%)	
Oral submucous fibrosis	6 (15%)	

Sample Site		
Buccal mucosa	31 (77.5%)	3 (30%)
Labial mucosa	7 (17.5%)	0
Tongue	2 (5%)	0
Alveolar mucosa	0	7 (70%)

Dysplasia		Not Applicable
Mild	7 (17.5%)	
Moderate	13 (32.5%)	
Severe	20 (50%)	

### Immunohistochemistry Findings

A comparison of ALDH1, SOX2, CD44, OCT4, and p16 immunoexpression for the OED and Normal groups is provided in Table 2. There was a significant difference in ALDH1 and SOX2 expression between the OED and Normal groups.

**Table 2:** Comparison of ALDH1, SOX2, CD44, OCT4, and p16 Immunoexpression between the OED and Normal Groups.

Marker	OED Group Score (Mean±SD)	Normal Group Score (Mean±SD)	*P* Value
ALDH1	5.00±3.84	0.62±1.24*	0.001
SOX2	4.42±3.81	1.62±2.05*	0.031
CD44	7.29±4.08	9.10±3.84	0.206
OCT4	2.15±2.55	3.09±3.54	0.410
p16	2.14±2.40	1.52±1.15	0.436

* *P*<0.05 significant when comparing the OED and Normal groups.

ALDH1, aldehyde dehydrogenase 1; CD44, cluster of differentiation 44; OCT4, octamer-binding protein 4; OED, oral epithelial dysplasia; SOX2, sex-determining region Y-box 2.

**Figure 1:**
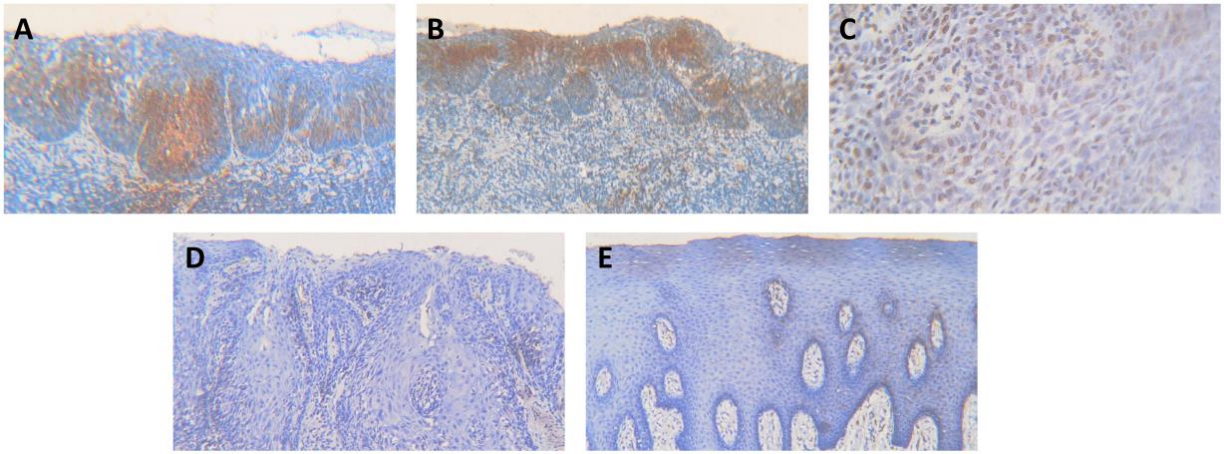
Photomicrographs of Immunohistochemical Staining in Severe P16-positive Oral Epithelial Dysplasia (OED) **A:** P16 positive staining. **B:** Positive ALDH1 staining. **C:** Positive OCT4 staining. **D:** Negative SOX2 staining. **E:** Negative CD44 staining. Original magnification ×100 for A, B, D, E; ×400 for C.

**Figure 2:**
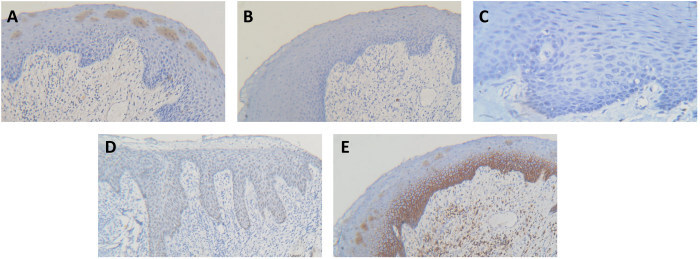
Photomicrographs of Immunohistochemical Staining in Mild P16-negative OED **A:** Patchy P16 staining. **B:** Negative ALDH1 staining. **C:** Negative OCT4 staining. **D:** Positive SOX2 staining. **E:** Positive CD44 staining. Original magnification ×100 for A, B, D, E; ×400 for C.

Figures 1 and 2 show the immunohistochemical expression of these markers in HPV-positive and negative OED, respectively. When the cases were stratified according to p16 positivity, none of the other markers were significantly different, although ALDH1 came close (Table 3).

**Table 3:** Comparison of ALDH1, SOX2, CD44, and OCT4 Values between p16-positive and p16-negative Oral Epithelial Dysplasia.

Observation	p16-positive (Mean±SD)	p16-negative (Mean±SD)	*P* Value
ALDH1	6.92±3.67	4.36±3.73	0.068
SOX2	3.86±4.13	4.61±3.75	0.597
CD44	6.47±3.61	7.56±4.25	0.470
OCT4	2.97±3.84	1.88±1.96	0.249

ALDH1, aldehyde dehydrogenase 1; CD44, cluster of differentiation 44; OCT4, octamer-binding protein 4; SOX2, sex-determining region Y-box 2.

## DISCUSSION

Scrutinizing the etiology or risk factors for initiation and development of malignancy has always been a challenge. Though tobacco and alcohol consumption are thought to be the most common risk factors for developing HNSCC, viral infections have also been indicated to play an important role in malignancy.^30^,^31^ The role of HPV in oral and oropharyngeal carcinoma was first proposed by Syrjänen et al. in 1983 and subsequently favored by others.^31^–^34^ Several different studies have indicated that overexpression of the p16INK4A protein by IHC can serve as a surrogate biomarker of HPV-induced carcinomas.^34^–^36^ However, the results vary for p16 expression in OED. Saito et al. reported p16 positivity in only 12% of 57 OED cases.^37^ In contrast, the study by Buajeeb et al. found no p16 positivity in oral leukoplakia cases with dysplasia.^38^ The present study reported 25% (10/40) of p16 positivity in the OED cases examined. High-risk HPV-positive OED has been reported to show strong p16 expression from half to full thickness.^29^,^38^,^39^ Similar to the present study, OED cases showed positive p16 expression from half to the full epithelial thickness in moderate and severe dysplasia. Likewise, the present study found that p16 overexpression was positively correlated with high-grade (moderate/severe) OED, similar to the observations reported by Cunningham et al.^36^ Angiero et al. reported an absence of p16 in normal mucosa.^40^ The present study also reported an absence of p16 immunoexpression in normal mucosa. Nevertheless, the presence of p16 throughout the epithelial transformations from mild to severe dysplasia suggests that viral infection is an important and independent event necessary for malignant transformation.^40^ The p16 IHC analysis is technically simple and appears to be a reliable indicator of HPV-associated high-grade oral squamous dysplasia.^36^ However, no definitive conclusion may be drawn from our findings on the causal relationship between p16 immunopositivity and the development of dysplasia.

Cancer stem cells are a rare subset of cancer cells with self-renewing ability. Investigation of CSC markers and targeting them selectively is the focus of many types of cancer research, including for OSCC. The use of biomarkers to investigate the early stages of OSCC could lead to the development of preventive therapeutic approaches to control the disease in the primary phase.^41^ But studies recording CSC marker expression in HPV-induced OED are relatively scarce in the literature. Hence this study attempted to find the correlation between putative stem cell markers and HPV status in OED using p16 immunostaining. To the best of our knowledge, this is the first study to examine the relationship between the putative CSC markers and p16 in OED in the English literature.

The level of ALDH1 activity can define normal tissue stem cells and CSC populations, where it is involved in self-renewal, differentiation, and self-protection. Accumulating evidence suggests that ALDH1 may represent a useful therapeutic CSC target in tissues not normally expressing high levels of ALDH1.^42^ Considering that this protein is not present at high levels in the oral mucosa, ALDH1 may represent a therapeutic opportunity for preventing the progression to oral cancer in patients with dysplasia.^43^ In the present study, 67.5% of OED cases had positive ALDH1 expression, unlike the findings of Liu et al. and Visus et al. where ALDH1 expression was found in only 38.3% and 32.5% of OED patients, respectively.^44^,^45^ This suggests the role of ALDH1 in the stepwise transformation of OED to carcinomas, since various studies have suggested that ALDH1 expression is a predictive marker for the malignant transformation of OED.^46^ However, the present study found no statistically significant correlation in ALDH1 and p16 expression, even though the mean value of ALDH1 immunoexpression was higher in p16-positive cases.

The transmembrane surface glycoprotein CD44 normally functions as an adhesion molecule through interactions with hyaluronan and cytoskeletal components and maintains tyrosine kinase activity. Changes in CD44 expression have been correlated with poor prognosis in several human malignancies.^46^ Some studies have reported that epithelium expressing CD44, under normal conditions such as squamous epithelium of the skin, endometrium, urothelium, laryngeal mucosa, and oral mucosa, has a tendency for downregulation during turnout progression and metastatic spread.^47^ Bahar et al. reported that, in dysplasia, 78% of cases showed a tendency toward downregulation of expression, which correlated to the degree of dysplasia.^49^ In the present study, the mean of CD44 immunopositive cells was higher in normal mucosa as compared to OED. The correlation between the degree of dysplasia and CD44 downregulation might reflect early cellular changes from normal cell–cell and cell–matrix interactions toward the bizarre, pathophysiological heterotypic cell surface adhesion property, which may contribute to cell invasion and the early development of malignant tumors in the oral cavity.^47^,^48^ Bolger et al. reported that HPV downregulates the stem cell marker CD44 in viral-related OED, with p16-positive OED being significantly lower for CD44 membranous immunoexpression than p16-negative cases.^49^ This finding is consistent with the results of the present study that showed increased CD44 expression in p16-negative cases.

The markers SOX2 and OCT4 are shown to act as important transcriptional factors to maintain the self-renewal capability of embryonic stem cells. They have been demonstrated to be good indicators of stem cell capacity in CSCs.^50^ Studies recording SOX2 and OCT4 expression have shown varying results in the literature. In the present study, the increase in mean SOX2 expression from normal mucosa to OED was statistically significant, which is similar to the findings by Vijayakumar et al. and Verma et al.^50^,^51^ Though not statistically significant, SOX2 expression was found to be downregulated in more p16-positive cases than in p16-negative cases, similar to the findings observed for CD44 expression. Thus, SOX2 may be associated with tumorigenesis and may serve as a predictor of malignant transformation of high-risk OED.^52^ The expression of OCT4 in normal mucosa was higher (40%) when compared to OED cases (37.5%) in the present study. Similarly, Motahari et al. reported significantly lower expression of OCT4 in OED than in normal oral epithelium.^53^ However, in a study by Qiao et al., the percentage of OCT4 positivity was noted to be 70% in OED; this higher expression may be due to the cases selected in their study, as they included lichen planus along with leukoplakia for potentially malignant disorders.^54^ In contrast to SOX2, the expression of OCT4 was increased in p16-positive cases, though it was not statistically significant (Table 3). This discrepancy in SOX2 and OCT4 expression is supported by the finding that while some subsets of stem cells may show both SOX2 and OCT4, others show SOX2 alone.^50^ In a study on three cancer cell lines, Cai et al. observed that, at the protein level, SOX2 may suppress further OCT4 expression via a positive loop, while downregulation of OCT4 could upregulate SOX2 expression via negative feedback in OCT4-low cancer cells.^55^ This finding was also validated by the observations of Vijayakumar et al., in which OCT4 was negative in most cases with high SOX2 expression.^50^ Similar findings were observed in p16-negative cases in the present study.

**Figure 3:**
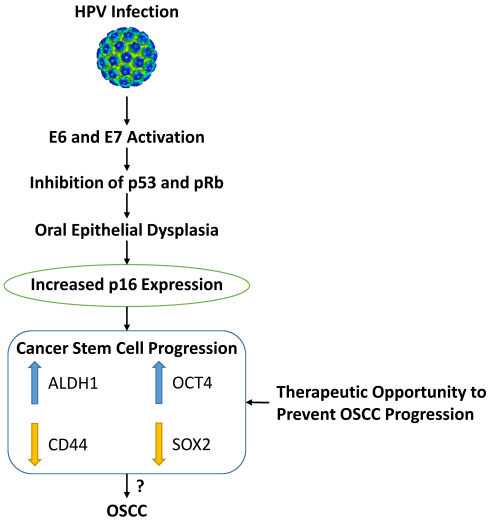
Schematic Representation of the Correlation between CSC and HPV-based Oral Cancer Initiation and Progression.

The present study showed that HPV upregulates ALDH1 and OCT4 in OED, which could be due in part to their increased expression in p16-positive cases. However, CD44 and SOX2 were downregulated by HPV, as demonstrated by their decreased expression in p16-positive cases. These findings imply a suggestive link between CSC markers and HPV in OED, and that p16 has a significant role in CSC progression in OED. Figure 3 provides a schematic representation correlating CSC and HPV-based oral cancer initiation and progression.

The major clinical implication of this study is that the combination of CSC markers and p16 immunoexpression in OED could be beneficial in identifying cases with a high risk of malignant transformation. Moreover, treatment modalities targeting CSC populations are currently of interest. Hence, these novel findings in HPV-induced OED could provide a basis for the development of newer treatment modalities targeting CSCs, the drivers of tumorigenesis. However, follow-up analysis with a large cohort of patients is needed to confirm this finding.

Treatment modalities targeting CSC populations are currently of interest. Kulsum et al. demonstrated that ALDH1A1 inhibition reduced the migration rate, self-renewal capacity, and tumorigenicity of cancer cells *in vitro*.^56^ Furthermore, an *ex vivo* study showed that ALDH1A1-specific inhibitor in combination with cisplatin significantly decreased cell proliferation as compared to individual treatment; NCT-501, a potent and selective theophylline-based inhibitor of aldehyde dehydrogenase 1A1 was used for ALDH1A1 inhibition.^56^ Thus, currently, an ALDH1A1-specific therapy with stem cell signaling pathway inhibitors and/or antibody-based therapy is expected for targeting CSC. It was also reported that in animal model studies, ALDH1A1^−/−^ mice are viable, which implies that ALDH1A1 inhibition might not damage normal tissue stem cells in ALDH1A1-targeted therapy for CSC elimination.^42^

The best method for HPV detection remains controversial, and a frequently recommended strategy is to use a combination of p16 immunohistochemistry followed by real-time quantitative polymerase chain reaction for high-risk HPV.^57^,^58^ Thus, further analysis with this modality is needed to validate the results obtained in the present study. Likewise, we acknowledge that our low study numbers are a potential limiting factor, and further studies with large sample sizes are required to fully define the functional roles of these biomarkers in HPV-induced oral cancer initiation and disease progression. Due to the rarity of HPV-associated OED, collaborative or multi-institution studies may help to overcome this limitation in the future.

## CONCLUSION

This study demonstrated a correlation between HPV status and CSC expression in OED and that the combined immunoexpression of p16 and CSC markers may improve our ability to predict OED outcomes. Future studies with a large cohort of patients and follow-up analyses are needed to validate these findings.

## Abbreviations

ALDH1: aldehyde dehydrogenase 1
CD44: cluster of differentiation 44
CSC: cancer stem cell
FFPE: formalin-fixed paraffin-embedded
HNSCC: head and neck squamous cell carcinoma
HPV: human papilloma virus
IHC: immunohistochemistry; min, minutes
OCT4: octamer-binding protein 4
OED: oral epithelial dysplasia
OSCC: oral squamous cell carcinoma
SOX2: sex-determining region Y-box 2
SRY: sex-determining region Y
